# Intermittent fasting in osteoarthritis: from mechanistic insights to therapeutic potential

**DOI:** 10.3389/fnut.2025.1604872

**Published:** 2025-07-21

**Authors:** Nianyi Sun, Yinuo Zhao, Junyu Wang, Anren Zhang, Yu He

**Affiliations:** ^1^Department of Rehabilitation, Shanghai Fourth People’s Hospital, School of Medicine, Tongji University, Shanghai, China; ^2^School of Medicine, Tongji University, Shanghai, China; ^3^Department of Rehabilitation, Shengjing Hospital of China Medical University, Shenyang, China; ^4^Engineering Research Center for Medical Surgery and Rehabilitation Robotics Technology of Liaoning Provincial, Shenyang, China

**Keywords:** osteoarthritis, intermittent fasting, metabolic regulation, inflammation, gut microbiota

## Abstract

Osteoarthritis (OA) is a prevalent whole-joint disease characterized by cartilage degradation, subchondral bone remodeling, synovial inflammation, and systemic metabolic dysregulation, imposing significant health and socioeconomic burdens globally. Conventional treatments primarily offer symptomatic relief without addressing the underlying disease mechanisms. Recently, intermittent fasting (IF), defined by cyclic caloric restriction and metabolic switching, has emerged as a promising lifestyle intervention with therapeutic potential for OA. Preclinical and preliminary clinical studies suggest that IF beneficially impacts OA pathogenesis by improving metabolic profiles, reducing systemic and local joint inflammation, activating cellular protective autophagy pathways, and positively modulating the gut microbiota. This review systematically synthesizes current mechanistic insights, preclinical findings, and emerging clinical evidence regarding IF’s role in OA prevention and treatment. We also address practical considerations for implementing IF in clinical practice and outline future research priorities necessary to validate and optimize IF protocols tailored for OA management.

## 1 Introduction

Osteoarthritis (OA) is a whole-joint disease characterized by structural alterations involving hyaline articular cartilage, subchondral bone, ligaments, the joint capsule, synovium, and periarticular muscles (1–3). According to the Global Burden of Disease study, OA is one of the leading causes of chronic pain and functional impairment among older adults, affecting approximately 595 million individuals worldwide (1), resulting in a significant public health and socioeconomic burden (4–6). Conventional therapies for OA primarily focus on symptom relief but fail to fundamentally reverse disease progression. Additionally, pharmacological interventions may present adverse effects, while joint replacement surgery is associated with high costs and postoperative risks (7, 8). Therefore, there is an urgent need to develop novel therapeutic strategies, especially those targeting early pathological changes in OA, aiming to halt or delay disease progression, enhance patients’ quality of life, and reduce long-term healthcare costs (1, 3, 9, 10).

In recent years, significant advancements have been made in understanding the etiology and pathogenesis of OA. The conceptual framework has gradually evolved from the traditional viewpoint of “pure mechanical wear” to a multifaceted interaction involving metabolic disturbances, inflammatory processes, and immune dysregulation, etc (11–14). Current studies indicate that obesity, insulin resistance, dysregulation of glucose and lipid metabolism, and chronic low-grade inflammation may accelerate the onset and progression of OA (1, 9, 15, 16). Extensive research supports dietary interventions as promising strategies for preventing or managing OA, highlighting their anti-inflammatory, antioxidant, and anabolic potentials, thus offering a feasible approach for reducing the disease burden (17).

Recently, intermittent fasting (IF) has attracted scholarly interest regarding its potential application in OA prevention and management (18). Studies suggest that IF, characterized by periodic restriction of caloric intake, induces metabolic switching, improves insulin sensitivity, reduces pro-inflammatory cytokine production, and positively modulates the immune microenvironment (19–22). These mechanisms collectively suggest a promising therapeutic potential for OA treatment and prevention (17, 23). As a lifestyle intervention affecting systemic metabolic and immune status, IF has the potential to shift the traditional treatment paradigm, which primarily focuses on local joint pathology, toward a broader, systemic modulation involving metabolism, inflammation, and gut microbiota (24).

This review focuses on recent advances concerning IF for OA, systematically summarizing the potential underlying mechanisms, evidence from animal studies and clinical trials, and discussing practical considerations and future research directions. We have searched the Pubmed and Embase databases to identify relevant literatures regardless of study type. To construct the search strategy, the concepts of “subjects: OA” and “intervention: IF” were combined with the “AND” operator. For each concept, we combined synonyms and Medical Subject Headings terms with the “OR” operator. In addition, we have handsearched the reference lists of relevant literatures to identify any related studies. Our aim is to provide a comprehensive and insightful overview of this emerging interdisciplinary field to support the scientific evaluation and practical application of IF as a non-pharmacological intervention for OA.

## 2 Overview of IF

### 2.1 Definition and common patterns

Intermittent fasting is a dietary pattern characterized by intentional restriction or complete cessation of energy intake during specific periods. Its fundamental principle involves triggering metabolic adaptations through cyclic periods of “feeding-fasting” (20, 25). IF has been demonstrated to provide numerous health benefits, such as weight loss (26, 27), regulation of body composition (28–30), reduction of inflammation (31, 32), alleviation of oxidative stress (33), improvement of insulin resistance (26, 34), promotion of neuronal regeneration and repair (35), acceleration of wound healing (36), and deceleration of cancer progression (37, 38). Commonly practiced IF patterns include (39):

• Alternate-day fasting (ADF): This regimen alternates between “fasting days” and “feast days”. On fasting days, individuals either consume only water (known as “zero-calorie alternate-day fasting”) (40) or restrict their caloric intake to approximately 25% of their daily energy requirements (around 500 kcal/day), termed “modified alternate-day fasting” (41). On feast days, there are no specific limitations regarding food types or quantities consumed.
• The 5:2 fasting diet: A modified form of ADF, this pattern involves normal eating for 5 days each week and caloric restriction to 500–600 kcal for the remaining 2 days (42).
• Time-restricted feeding (TRF): Unlike ADF and the 5:2 fasting diet, TRF restricts daily food consumption to a specific time window (43). Specifically, TRF limits the feeding period to certain hours each day (typically 4–8 h), with fasting (consumption limited to water or zero-calorie beverages) occurring during the remaining hours (30). As a distinct form of IF, TRF does not require individuals to monitor caloric intake or count calories during the eating window (44).

While these IF modalities differ in terms of fasting duration and frequency, they all aim to induce a state of energy deprivation, thereby promoting metabolic switching in the body (19, 45).

### 2.2 Physiological and metabolic basis of IF

The primary physiological mechanism underlying IF is known as “metabolic switching,” where the body transitions from primarily utilizing glucose as an energy source to predominantly relying on fatty acid oxidation and ketogenesis (20, 45). During fasting periods, hepatic glycogen reserves gradually become depleted, accelerating lipolysis and the subsequent generation of ketone bodies (e.g., β-hydroxybutyrate). These ketones serve not only as alternative energy substrates but also as signaling molecules that regulate inflammation, autophagy, and stress responses (19, 39). Ketone bodies produced during fasting significantly influence the expression and activity of various key proteins and molecules, including peroxisome proliferator-activated receptor γ coactivator-1α (PGC-1α), sirtuins, fibroblast growth factors, and nicotinamide adenine dinucleotide (NAD +), all of which play critical roles in health and aging (25). By interacting with these cellular pathways, ketone bodies exert profound systemic metabolic effects (36). Furthermore, IF may regulate cellular energy metabolism and oxidative stress by lowering insulin and insulin-like growth factor-1 (IGF-1) levels and activating signaling pathways such as AMPK and SIRT1 (21). Compared to continuous caloric restriction (CR), IF demonstrates advantages in preserving lean body mass and generally shows higher adherence rates (22, 46).

Research has indicated that IF not only promotes weight loss and reduces free radical generation but also induces adaptive cellular responses, enhancing stress resistance and suppressing inflammation (47). IF triggers coordinated adaptive stress responses at the cellular level, resulting in upregulation of antioxidant defenses, DNA repair, protein quality control, mitochondrial biogenesis, and autophagy, coupled with a reduction in inflammation (48, 49). Through intermittent metabolic switching, cellular and molecular mechanisms enhance organ functionality and improve resistance to stress and disease (25). During energy restriction, depletion of hepatic glycogen stores initiates metabolic switching, reducing fatty acid and ketone utilization (26). To accommodate this bioenergetic challenge, cells and organ systems activate multiple signaling pathways, which enhance mitochondrial function, boost stress resistance and antioxidant defense, diminish insulin signaling and global protein synthesis, and upregulate autophagy to clear damaged molecules and recycle cellular components (50–52). Conversely, during feeding periods, cells engage in tissue-specific growth and plasticity processes (53).

### 2.3 Association of IF with chronic disease management

Recently, IF has emerged as a popular strategy for managing various chronic diseases. Its extensive modulatory effects on metabolic pathways underscore its promise as a nutritional intervention to counteract obesity and associated metabolic disorders (48, 54). Studies suggest that IF holds potential therapeutic benefits for obesity, diabetes, cardiovascular diseases, and certain neurodegenerative conditions (19, 20, 55). Extensive evidence indicates that IF can decelerate cellular aging through multiple mechanisms, including the switching of metabolic rhythms to promote health span and mitigate age-related pathologies (56). Numerous clinical trials have demonstrated that IF restores metabolic homeostasis in patients with obesity and metabolic syndrome by ameliorating adiposity, lipid dysregulation and hypertension (57, 58). In the context of cardiometabolic disease, IF confers cardiovascular protection by lowering blood pressure, improving lipid profiles and reducing key atherogenic risk factors (59). Moreover, the enforced metabolic shift induced by IF may disrupt the vicious cycle of metaflammation, modulate cell- and tissue-specific immunometabolic responses, and reestablish endocrine control of energy balance (60).

As a non-pharmacological intervention, IF represents a potent tool in the comprehensive management of a wide spectrum of metabolic disorders. These chronic diseases share common pathological features of metabolic disturbances and chronic low-grade inflammation, which are also critical in the pathogenesis of OA. Consequently, increasing attention has been directed toward the application of IF in musculoskeletal disorders, particularly regarding its effects on bone and cartilage tissues (21, 61). Preliminary evidence indicates that IF might benefit bone and joint health by improving bone density and reducing inflammatory markers (39). Additionally, studies have shown that IF can alleviate symptoms of rheumatoid arthritis and spondyloarthritis (62–64), and significantly improve disease activity and symptoms such as dactylitis in patients with psoriatic arthritis, independent of weight loss. These beneficial effects might relate to IF’s immunomodulatory properties (65).

## 3 Osteoarthritis pathogenesis beyond mechanical stress

OA was traditionally regarded as a result of mechanical “wear and tear,” but recent research has demonstrated a more complex pathogenesis (10, 14). It typically involves cartilage, subchondral bone, and synovial tissue, each engaging in intricate cellular and molecular interactions (66). Damage to the cartilage extracellular matrix triggers the innate immune response through the release of damage-associated molecular patterns (DAMPs), resulting in the production of pro-inflammatory cytokines and matrix-degrading enzymes (67). Under metabolic stress, chondrocytes exhibit accelerated glycolysis, mitochondrial dysfunction, and impaired autophagy (68). Mild synovitis develops early in OA progression, where synovial immune cells produce inflammatory mediators such as interleukin-1β (IL-1β), tumor necrosis factor-alpha (TNF-α), and activated metalloproteinases (MMPs), further accelerating cartilage degradation (69). This creates a vicious cycle of inflammation and tissue damage (67). It is now widely recognized that OA is not restricted to the joint alone, but also involves systemic immune dysregulation that impacts the entire body (70).

### 3.1 Metabolic disorders

In addition to local joint mechanisms, systemic metabolic and inflammatory states significantly influence OA pathogenesis, particularly evident in obesity-associated OA. Epidemiological evidence shows that obesity substantially increases the risk of knee OA (KOA), and even non-weight-bearing joints (e.g., finger joints) are more commonly affected in obese individuals (71, 72). These findings suggest that obesity promotes OA through systemic metabolic and inflammatory pathways, beyond mere mechanical loading. Adipose tissue hypertrophy leads to macrophage infiltration and secretion of inflammatory cytokines such as TNF-α and IL-6, initiating systemic chronic low-grade inflammation (71, 73–75). These inflammatory mediators infiltrate the joint via synovial fluid, exacerbating cartilage degradation and synovial inflammation (76). A meta-analysis has revealed that circulating adiponectin concentrations are significantly elevated OA patients compared to healthy controls (77). Additionally, elevated leptin levels in obesity promote local joint inflammation and cartilage matrix degradation, indicating leptin as a critical mediator linking obesity and OA (71). Leptin, acting either autonomously or synergistically with other pro-inflammatory mediators, directly targets chondrocytes, synoviocytes and osteoblasts to profoundly influence the progression of osteoarthritis (78, 79). Inflammatory and catabolic factors such as IL-1β, matrix metalloproteinase-9 (MMP-9) and MMP-13 have been shown to upregulate leptin expression within joint tissues (80, 81). Elevated leptin levels, in turn, skew immune responses toward a T helper 1 (TH1) cytokine profile while suppressing TH2 cytokines (82). This shift exacerbates intra-articular inflammation and accelerates cartilage matrix degradation, thereby hastening disease progression (83–85). Notably, obese animal models deficient in leptin signaling do not develop systemic inflammation or KOA, highlighting leptin’s pivotal role in osteoarthritic pathobiology (86). Insulin resistance and hyperinsulinemia further exacerbate OA progression, with elevated insulin levels inducing abnormal chondrocyte differentiation and cartilage hypertrophy, thus accelerating cartilage degeneration (18). Under insulin-resistant conditions, articular chondrocytes exhibit reduced sensitivity to insulin, resulting in disrupted intracellular signaling that impairs cartilage matrix synthesis and repair (87). Chronic hyperglycemia drives the accumulation of advanced glycation end products (AGEs) within chondrocytes, engagement of AGEs with their cell-surface receptors activates pro-inflammatory pathways -most notably NF-κB- thereby promoting chondrocyte apoptosis and matrix breakdown (88, 89). Moreover, diabetic microvascular complications compromise subchondral perfusion, diminish nutrient exchange and alter synovial fluid composition, collectively accelerating cartilage degeneration (90).

Fatty acid metabolism dysregulation also plays a pivotal role in OA progression (91). Fatty acids, essential for cellular energy metabolism, significantly influence immune cell function and bone metabolism (92, 93). Recent studies indicate that dysregulated fatty acid metabolism in subchondral bone could trigger local metabolic imbalances, enhancing immune cell activation and inflammatory responses (94). For example, abnormal fatty acid metabolism in KOA could stimulate macrophages and T cells to secrete pro-inflammatory cytokines, exacerbating cartilage damage and bone loss (95). Investigations have consistently demonstrated a positive association between hypercholesterolemia and OA (96, 97). Elevated plasma cholesterol levels inhibit LRP3 expression in chondrocytes, disrupting extracellular matrix turnover and precipitating cartilage degeneration (98). Concomitantly, heightened circulating cholesterol and triglycerides, along with dysfunctional high-density lipoprotein, drive ectopic ossification and bone-marrow lesion formation—mechanisms that exacerbate cartilage loss in KOA and accelerate joint pathology (99, 100). Moreover, metabolic syndrome contributes to osteoarthritic pain through non-mechanical pathways, including systemic inflammation, dysregulated endocannabinoid signaling, transient receptor potential vanillic acid subfamily member 1 (TRPV1) channel activation, and gut microbiota imbalances, further underscoring lipid metabolism’s pivotal role in OA pathogenesis (101, 102).

Moreover, metabolic dysregulation not only perturbs chondrocyte bioenergetics but also inflicts direct damage through mechanisms such as inflammation and oxidative stress, leading to chondrocyte apoptosis and dysfunction that exacerbate cartilage degeneration (103, 104). Studies have shown that diet-induced obesity is associated with elevated levels of inflammatory cytokines, including leptin and IL-1α, which correlate with the progression of OA, underscoring metabolic abnormalities’ role in driving inflammatory cascades that exacerbate joint pathology (105). Consequently, OA is increasingly considered a joint manifestation of metabolic syndrome, suggesting metabolic modulation could influence OA progression.

### 3.2 Chronic inflammation

Although OA was historically perceived primarily as a degenerative condition, recent studies emphasize the crucial role of chronic inflammation in OA progression (70, 106). Another hallmark of inflammation is the chronic activation of the immune system: senescent immune cells acquire a pro-inflammatory phenotype, while microbial infection or gut dysbiosis can both initiate and modulate this persistent inflammatory state (107). Damage-associated molecular patterns (DAMPs) significantly contribute to systemic inflammation and joint destruction. Mitochondrial stress responses and dysregulated glucose and lipid metabolism also promote DAMP production (108). DAMPs exacerbate joint tissue damage by activating immune cells, promoting inflammation, inducing cartilage matrix degradation, and triggering chondrocyte apoptosis (109, 110). Increased cytokines and chemokines within OA joints activate NLRP3 inflammasomes in chondrocytes and synoviocytes, further accelerating joint destruction (21). Persistent low-grade inflammation in OA is in part driven by sustained activation of signaling cascades such as NF-κB and mitogen-activated protein kinase (MAPK) (111). As a master transcriptional regulator of the inflammatory response, NF-κB is activated by diverse pro-inflammatory stimuli and orchestrates the expression of cytokines and MPPs (112). Chronic inflammation also induces synovial hyperplasia and leukocyte infiltration, culminating in synovitis that exacerbates joint inflammation and tissue damage (113, 114). Moreover, the inflammatory milieu perturbs bone remodeling, skewing the balance toward osteoclast-mediated resorption over osteoblast-driven formation and thereby accelerates structural joint deterioration (115, 116).

### 3.3 Oxidative stress and mitochondrial dysfunction

Mitochondrial dysfunction is characterized by mitochondrial DNA damage, decreased mitochondrial membrane potential, reduced oxidative phosphorylation efficiency, and increased reactive oxygen species (ROS) production. Collectively, these alterations lead to impaired mitochondrial function, negatively affecting normal cellular metabolism and function (117). Oxidative stress arises from an imbalance between ROS production and the cellular antioxidant defense capacity, playing a pivotal role in OA pathogenesis (118). Elevated oxidative stress observed in OA joints contributes significantly to chronic inflammation and results from excessive ROS production (69). Furthermore, mitochondrial damage activates the NOD-like receptor family pyrin domain-containing 3 (NLRP3) inflammasome, which triggers excessive secretion of inflammatory mediators such as IL-1β and TNF-α, exacerbating chondrocyte inflammation and promoting apoptosis (119). Mitochondrial dysfunction not only amplifies cartilage degradation by activating inflammatory signaling cascades and upregulating matrix metalloproteinases, but also compromises chondrocyte bioenergetics and survival through impaired adenosine triphosphate (ATP) synthesis and disrupted calcium homeostasis (120, 121). Research has shown that excessive generation of ROS establishes a deleterious positive feedback loop wherein ROS-induced mitochondrial damage further amplifies ROS production, which plays a key driving role in the pathogenesis of OA (122). Specifically, elevated ROS levels compromise mitochondrial integrity, leading to increased mitochondrial ROS (mROS) generation and establishing a self-amplifying cycle of oxidative damage and organelle dysfunction. This vicious feedback loop perpetuates mitochondrial impairment, exacerbates chondrocyte injury, and accelerates OA pathology (123). In addition, oxidative stress also inhibits mitochondrial autophagy, making it difficult for damaged mitochondria to be cleared in a timely manner, further exacerbating mitochondrial dysfunction and chondrocyte damage, ultimately promoting the pathological process of OA (124, 125).

### 3.4 Autophagy dysfunction

Autophagy dysfunction plays a crucial role in OA pathogenesis (126). It prevents efficient clearance of damaged organelles and proteins, leading to cellular homeostasis disruption, accelerated chondrocyte senescence, and apoptosis. Autophagy impairment also exacerbates inflammation and cartilage matrix degradation (127). Autophagy can regulate the function of immune cells in joints by reducing the secretion of pro-inflammatory cytokines and inhibiting the inflammatory process of OA (128, 129). In bone cells, autophagy also plays an important role in regulating the balance between bone formation and resorption, ensuring normal bone metabolism (130). Dysfunction of autophagy may lead to abnormal bone cell function, which in turn affects the overall health of bones (131). Dysfunctional autophagy is closely linked to aberrant activation of signaling pathways, including excessive mTOR activation, suppressed AMPK signaling, and disruption of Beclin1 and LC3 pathways (132). Targeting these autophagy pathways may offer potential strategies for OA prevention and treatment (133, 134).

### 3.5 Gut microbiota dysbiosis

Gut microbiota dysbiosis refers to the disruption of intestinal microbial equilibrium caused by external factors such as diet, infections, aging, and genetics. This disruption leads to alterations in the structure, function, and diversity of gut microbiota, consequently resulting in various pathological conditions and diseases (135). Obesity and high-fat diets have been recently implicated in gut dysbiosis, potentially influencing OA development through the gut-joint axis (24, 136, 137). Dysbiosis of the intestinal microbiota contributes to systemic inflammation and immune dysregulation, ultimately affecting cartilage and the joint microenvironment (138). High-fat diets increase the proportion of Gram-negative bacteria in the gut, enhancing the production of endotoxins such as lipopolysaccharides (LPS), which increase intestinal barrier permeability and lead to leakage of inflammatory metabolites like LPS into systemic circulation, thereby inducing systemic low-grade inflammation (137, 139). Elevated LPS levels activate the TLR4 pathway, promote M1 macrophage polarization, release pro-inflammatory cytokines, and intensify tissue inflammatory damage (140). Additionally, beneficial gut bacteria produce extracellular vesicles transported to host bone cells, participating in the regulation of bone metabolism (141). In a rat model of high-fat, high-sucrose diet-induced obesity, gut microbiota dysbiosis was closely linked to elevated systemic LPS levels, enhanced serum and local inflammatory signatures, and aggravated joint damage (142). Mouse studies have demonstrated that obesity induced by a high-fat diet can alter the Firmicutes/Bacteroidetes ratio, promoting inflammatory bacteria growth, elevating circulating LPS levels, and exacerbating joint inflammation (143). Conversely, prebiotic fructooligosaccharides, which modulate gut microbiota, reduce systemic inflammation in obese mice and significantly mitigate cartilage damage in traumatic knee OA (71). These findings suggest that targeting gut microbiota to modulate inflammation represents a promising new intervention strategy for OA (24). Emerging evidence indicates that gut microbiota modulate joint immunity by regulating immune cell differentiation and function (144, 145). Specific microbial taxa have been shown to skew T cell polarization toward the pro-inflammatory Th17 phenotype, thereby increasing IL-17 production and amplifying joint inflammation (146). Concurrently, dysbiosis may disrupt B cell homeostasis, leading to aberrant autoantibody generation and exacerbation of joint tissue damage (147). Furthermore, microbial metabolites such as short-chain fatty acids (SCFAs), notably butyrate, exert anti-inflammatory effects, potentially suppressing joint inflammation by enhancing regulatory T-cell activity, highlighting their crucial role in mediating gut microbiota effects on joint health (24).

**FIGURE 1:**
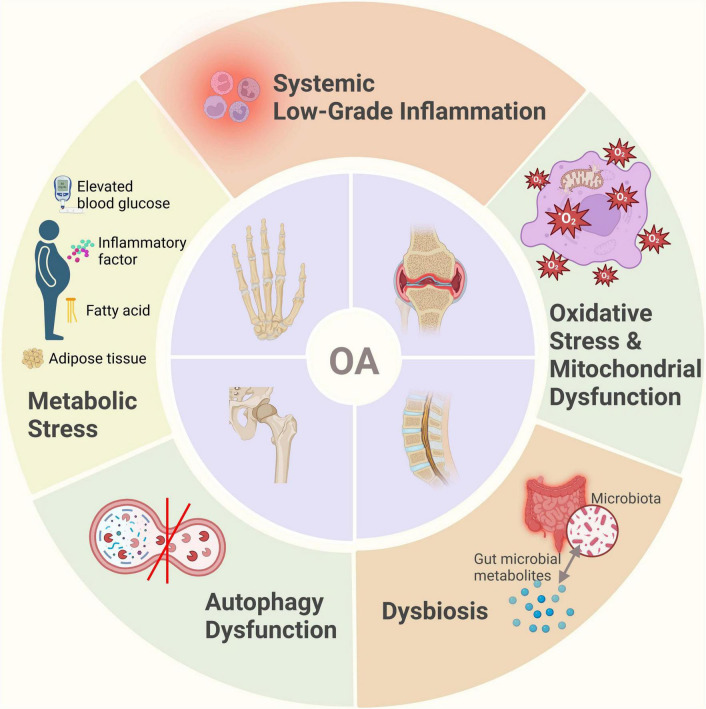
Schematic representation of OA pathogenesis beyond mechanical stress.

**FIGURE 2:**
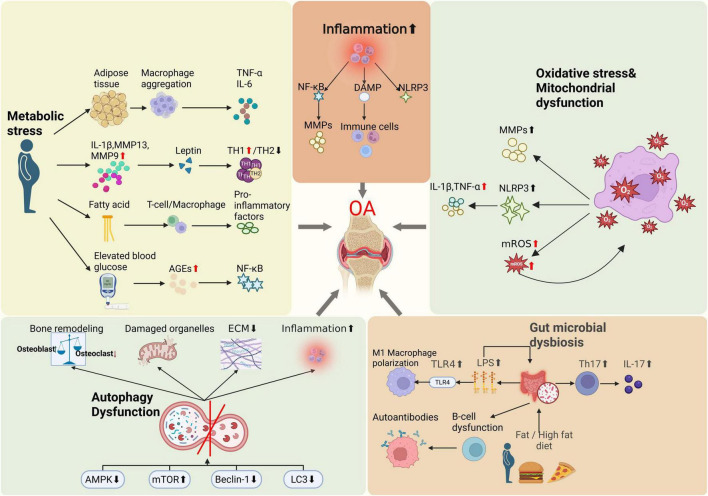
Illustrations of OA pathogenesis beyond mechanical stress.

In summary, the development of OA is not limited to local joint pathology but results from complex interactions involving systemic metabolism, immune regulation, and gut microbiota, among other factors (Figures 1, 2). This understanding provides a theoretical foundation for systemic interventions in OA management, such as dietary modulation.

## 4 Potential biological mechanisms of IF in the prevention and treatment of OA

### 4.1 Improvement of metabolic markers and cartilage protection

IF induces periodic low-energy intake, driving a metabolic switch that enhances lipolysis and ketone body production (20). In the osteoarthritic joint environment, improving chondrocyte energy homeostasis supports the synthesis and repair capacity of the cartilage extracellular matrix. Activation of the AMPK/SIRT1 axis promotes mitochondrial biogenesis and autophagy, while concurrently suppressing catabolic mediators such as hypoxia-inducible factor 2α (HIF-2α), thereby slowing cartilage degeneration (19). Additionally, IF increases free fatty acid (FFA) levels, leading to elevated production and release of fibroblast growth factor 21 (FGF21) (63). Evidence indicates that FGF21 not only regulates glucose and lipid metabolism but may also significantly influence cartilage metabolism and inflammatory responses, thereby impacting OA onset and progression (148). Moreover, IF-induced reductions in insulin and insulin-like growth factor-1 (IGF-1) concentrations exert protective effects on cartilage. Elevated insulin and IGF-1 conditions can disrupt chondrocyte metabolism, potentially accelerating OA progression (18, 149, 150). IF enhances insulin sensitivity, reducing fasting insulin levels and thereby mitigating hyperinsulinemia. It has been reported (18) that elevated fasting insulin in OA patients may exacerbate cartilage matrix degradation, whereas IF can lower fasting insulin by approximately 20%–30%, thus reducing metabolic stress on cartilage. Furthermore, IF typically improves lipid profiles-especially reducing triglycerides and low-density lipoprotein (LDL)-and beneficially modulates adipokines by decreasing leptin and increasing adiponectin levels (63). These metabolic adjustments may alleviate the detrimental metabolic load on articular cartilage and synovium (151). For instance, decreased leptin levels reduce stimulation of MMP production in chondrocytes, while increased adiponectin exerts anti-inflammatory effects and promotes matrix synthesis (152). It was reported that (25, 79, 153) serum leptin levels in KOA patients correlated with pain severity and structural damage, and dietary interventions lowering leptin may slow cartilage degeneration to some extent. Thus, through multilayered metabolic improvements (e.g., reduced insulin and leptin, improved lipid profiles), IF may foster a systemic metabolic environment that is beneficial to joint health, ultimately delaying OA progression.

### 4.2 Anti-inflammatory effects and immunomodulation

Metabolic changes induced by IF directly reduce systemic inflammation. Under fasting conditions, metabolites such as β-hydroxybutyrate (BHB) are produced, which have demonstrated inhibitory effects on inflammatory pathways including the NLRP3 inflammasome (18). Clinical studies have consistently shown that various forms of IF effectively decrease chronic inflammatory biomarkers such as C-reactive protein (CRP), TNF-α, and IL-6 (154–156). For OA, these changes imply that IF can alleviate local joint inflammation. A systematic review focused on chronic pain noted that in OA patients, IF-induced fat loss reduces adipose-derived pro-inflammatory cytokines, consequently lowering inflammation both systemically and within joint tissues (157). Additionally, IF may decrease the proportion of pro-inflammatory synovial macrophages and enhance peripheral regulatory T-cell populations, thus improving immune balance (158). In rheumatoid arthritis (RA) studies, patients undergoing strict fasting for seven or more days experienced significant reductions in joint swelling and tenderness, along with notable alterations in serum IL-6 and IL-1 receptor antagonist concentrations (18). Although OA inflammation is typically less severe compared to RA, chronic low-grade inflammation persists throughout the OA disease process (14). IF exerts its anti-inflammatory effects through multiple mechanisms: firstly, by reducing visceral adiposity and gut-derived inflammatory mediators; secondly, by directly modulating cellular metabolic pathways, leading to decreased expression of pro-inflammatory genes, thereby potentially attenuating the inflammatory drivers of OA (20). Furthermore, IF-mediated suppression of NF-κB and NLRP3 inflammasome pathways contributes significantly to decreased inflammation both locally in joints and systemically (159). IF may also modify adipokine secretion profiles and regulate immune cell functions and pro-inflammatory cytokine networks, thus alleviating synovial inflammation (160, 161). Of note, recent discoveries regarding the neuropeptide Y (NPY) pathway offer novel insights into the mechanisms underlying IF’s anti-inflammatory effects in OA. New animal studies suggest that osteocytes overproduce NPY during OA progression, promoting inflammation and osteoclastogenesis, whereas IF significantly inhibits osteocytic NPY overproduction (162). Further experiments indicated that osteocyte-specific NPY knockout markedly attenuated surgically-induced OA inflammation and bone destruction, and notably, the protective effects of IF against OA were abolished in the absence of NPY. These findings strongly suggest that IF may exert its anti-inflammatory and bone-protective effects via downregulation of osteocyte-derived NPY, establishing the NPY pathway as an innovative key mechanism by which IF impacts OA pathogenesis.

### 4.3 Autophagy activation and cellular protection

Autophagy is an essential cellular self-protective mechanism that plays a crucial role in maintaining the homeostasis of chondrocytes and the cartilage extracellular matrix (163). The aging and cell death observed in osteoarthritic cartilage are closely associated with impaired autophagic activity (164, 165). Under physiological conditions, chondrocytes depend on moderate autophagy to eliminate damaged organelles; however, the expression of autophagy-related proteins, such as Beclin-1 and LC3, is diminished in aged and osteoarthritic cartilage. This reduction results in the accumulation of intracellular damaged products and decreased chondrocyte viability (166, 167). Fasting activates autophagy by restricting energy intake and plays a significant role in regulating mitochondrial network homeostasis (168). IF is widely recognized as a robust inducer of autophagy: during fasting periods, activation of the energy-sensing pathway AMPK and inhibition of mTOR enhance autophagosome formation (45). A recent study by Zhang et al. (23) underscored the importance of IF-induced autophagy in protecting cartilage. They demonstrated that IF significantly activates selective mitophagy in chondrocytes, which facilitates the clearance of damaged mitochondria, reduces oxidative stress and apoptosis, and ultimately prevents OA progression in mouse models. Specifically, Optineurin (Optn) gene knockout mice exhibited accelerated cartilage degeneration and OA phenotype, whereas IF treatment restored mitophagy through the OPTN pathway and significantly alleviated cartilage lesions. These findings suggest that maintaining chondrocyte autophagy is crucial for combating OA, and IF provides a systemic means to enhance autophagic activity. In addition to chondrocytes, IF also modulates autophagy in other joint cells. For example, IF can induce autophagy in synovial fibroblasts, thereby suppressing synovial hyperproliferation and inflammatory mediator release (63, 64). Furthermore, IF may promote macrophage polarization toward the anti-inflammatory M2 phenotype, a process closely linked with enhanced autophagy (158). Taken together, IF exerts protective effects at the cellular level by activating autophagy, facilitating the clearance of damage-associated factors in degenerative tissues, and counteracting age- and inflammation-related damage, ultimately slowing the pathological progression of OA (33).

### 4.4 Gut microbiota-joint axis

The impact of IF on gut microbiota represents a crucial aspect of its underlying mechanisms (45). Fasting-feeding cycles can alter nutrient availability patterns within the gut, thus reshaping microbial composition and the profile of microbial metabolites (169, 170). Recent studies have demonstrated that IF enhances gut microbial diversity, increases beneficial bacterial populations, promotes SCFAs production, and concurrently reduces pro-inflammatory bacteria and harmful metabolites (171, 172). For instance, a study conducted in hypertensive rats found that ADF increased the abundance of beneficial microbes, such as Bifidobacterium, and elevated bile acid-related microbial metabolites, which contributed to reduced blood pressure and systemic inflammation (158). In obese individuals, an 8-week intervention combining IF with a high-protein diet significantly enriched Christensenellaceae in fecal microbiota-a bacterial family associated with healthy, lean phenotypes (173). These microbiota changes correlated with improved gastrointestinal symptoms and reduced systemic metabolic inflammation markers. IF has also been suggested to exert anti-inflammatory and neuroprotective effects by modulating gut microbiota (48). Animal studies further indicated that IF partially restores gut microbiota balance in obesity models, alleviating inflammatory burden and slowing joint degeneration (19). The beneficial effects of improved gut microbiota composition on OA may be explained by several mechanisms. Enhanced gut barrier integrity, due to increased abundance of butyrate-producing bacteria and probiotics, reduces translocation of inflammatory molecules like LPS (137). Additionally, SCFAs such as butyrate and propionate, produced by beneficial bacteria, enter systemic circulation and can directly inhibit joint inflammation and enhance chondrocyte energy metabolism (24). Furthermore, IF may modulate gut immune environments by increasing anti-inflammatory regulatory T cells and reducing pro-inflammatory Th17 cells within gut-associated lymphoid tissues, thereby alleviating systemic inflammation (170). Although direct evidence linking IF, gut microbiota, and OA remains limited, considering the crucial role of gut microbiota in obesity and inflammation, IF-mediated reshaping of the gut-joint axis may represent a novel therapeutic strategy for reducing joint inflammation induced by gut dysbiosis and ultimately preventing OA progression.

**FIGURE 3:**
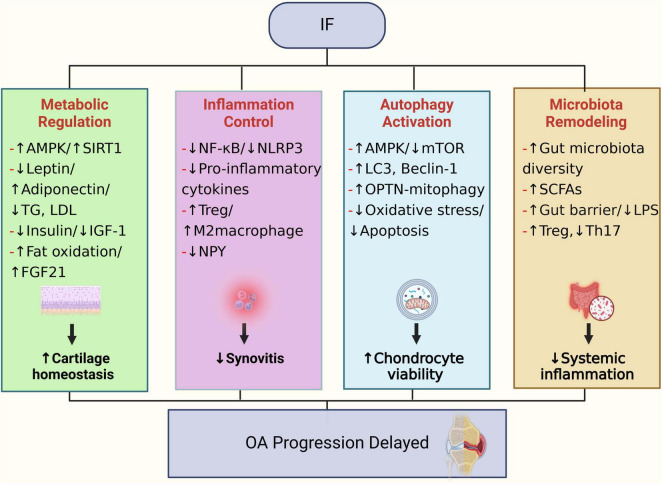
Potential biological mechanisms through which IF may contribute to the prevention and treatment of OA.

In summary, IF may confer beneficial effects on OA through multiple mechanisms, including metabolic optimization, anti-inflammatory immunomodulation, autophagy activation, and gut microbiota regulation (Figure 3).

## 5 Preclinical evidence

### 5.1 *In vivo* studies

To investigate the therapeutic effects of IF on OA, various animal model studies have been conducted. Qian et al. (162) employed a mouse model of destabilization of the medial meniscus (DMM)-induced KOA. After surgery for 1 week, these mice or the sham-operated mice received one, two, four, or six cycles of IF (ADF for 1 week and then refeeding for another week) or *ad libitum* feeding. They observed that IF treatment effectively preserved subchondral bone microarchitecture and attenuated cartilage degeneration. Moreover, IF-treated mice exhibited significantly improved outcomes in gait analysis, rotarod performance, balance beam tests, and pain sensitivity assays compared to controls, suggesting that IF enhances joint function and alleviates pain perception in OA animals. These findings provided initial evidence that IF could mitigate structural deterioration and symptoms associated with OA. Importantly, the study identified NPY as a crucial mediator of IF’s protective effects. Increased NPY expression was detected in the bone tissues of OA mice, whereas IF significantly downregulated osteocytic NPY levels. Furthermore, IF’s beneficial effects on OA were diminished in osteocyte-specific NPY-knockout mice, with some parameters even showing a slight worsening trend. These observations highlight NPY’s pivotal role in the IF-mediated alleviation of OA pathology. Thus, by reducing osteocytic NPY expression, IF effectively suppresses pathological processes such as inflammation, osteoclastogenesis, and neurogenic fiber proliferation in OA, representing a novel finding that emphasizes the essential role of bone tissues in IF-induced joint protection. Another notable study by Zhang et al. (23) focused on IF-mediated mitochondrial autophagy (mitophagy) in chondrocytes. Using Optineurin (Optn)-knockout mice to model impaired autophagy, the researchers demonstrated that Optn deficiency exacerbated chondrocyte injury and OA-like pathology. The mice were fasted for 24 h thrice a week (on days 2, 4, and 6), followed by *ad libitum* re-feeding of a standard solid diet for 1 week. This fasting-refeeding cycle was repeated for 2 months. ADF significantly activated OPTN-dependent mitophagy in wild-type OA mice, leading to efficient clearance of damaged mitochondria, and subsequently suppressed cartilage degeneration and joint inflammation. However, IF failed to prevent OA progression in Optn-deficient mice, indicating that the protective effect of IF on cartilage is primarily mediated via the OPTN-associated mitophagy pathway. Histological analyses revealed higher proteoglycan content, lower chondrocyte apoptosis rates, and reduced oxidative stress markers in synovial and subchondral bone tissues following IF treatment. These findings provide robust preclinical evidence demonstrating that autophagy activation is a critical mechanism underlying IF’s anti-OA effects. Collectively, these two animal studies support the multifaceted benefits of IF in OA models, including attenuated inflammation, reduced osteoclast activity, and cartilage protection, consistent with previously discussed mechanistic analyses. Additionally, some relevant investigations on IF in metabolic OA contexts are noteworthy. For instance, Park and Shin (174) studied a KOA rat model accompanied by obesity. In the IF groups, rats had a high-protein or high-fat diet for 3h at the beginning of the dark cycle (19.00 to 22.00 h), the mealtime of the rats corresponded to the morning for humans. The 3-h feeding period is similar to the 16-h fasting and 8-h feeding for humans in their previous and preliminary studies. Mimicking postmenopausal metabolic conditions and introducing Alzheimer’s-like pathological states to exacerbate systemic inflammation, their study revealed that a high-protein diet combined with IF effectively decreased body fat mass, increased lean body mass, and simultaneously reduced the joint expression of inflammatory cytokines such as IL-1β and TNF-α without significantly reducing total caloric intake. Histological examination of joint tissues indicated less cartilage erosion and bone resorption in IF-treated animals compared to controls. These findings suggest that dietary composition adjustments—particularly increased protein intake—may enhance the beneficial effects of IF on OA, notably by preventing muscle loss. This provides valuable guidance for clinical practice, indicating that older OA patients employing IF should ensure adequate protein consumption to optimize outcomes between weight loss and muscle preservation.

Glucosamine (GlcN) is widely used to alleviate symptoms of osteoarthritis; however, its clinical efficacy is limited by poor bioavailability (175, 176). A recent animal study (177) investigated strategies to enhance GlcN absorption by leveraging circadian rhythms and feeding patterns (although not in OA models). The findings demonstrated that administering GlcN during the peak expression of its intestinal transporters (22:00) under fasting conditions significantly increased its systemic bioavailability. These insights suggest a promising chronopharmacological approach to optimize GlcN delivery in humans, potentially improving therapeutic outcomes for OA and related disorders.

### 5.2 *In vitro* studies

A study showed that the fasting mimicking diet can significantly improve the mitochondrial function of osteoblasts, enhance the regenerative ability of osteoprogenitors, aid in tissue repair, and regulate the expression of related genes. This discovery provides a new perspective for understanding the role of fasting in bone health (61). Another *in vitro* study (162) reported that NPY significantly promoted inflammatory cytokine expression in monocyte/macrophage populations, facilitated osteoclast differentiation, and enhanced neuronal neurite outgrowth. IF effectively inhibited these NPY-mediated pathological processes by reducing NPY expression levels in osteocytes, further elucidating the potential mechanisms underlying IF-mediated relief of OA symptoms.

There are research results showing that (178) in food-restricted mice, the expression of fibroblast growth factor 21 (FGF21) in the tibial growth plates is greater than that in mice fed randomly. FGF21 has a pathogenic effect of inhibiting bone growth during long-term malnutrition. Another study on the same team showed that (179) fasting would induce an increase in the expression of FGF21, and the increase in FGF21 would inhibit the proliferation and differentiation of chondrocytes, reduce the binding of growth hormone to chondrocytes, and have a significant inhibitory effect on bone growth. However, there are also experiments showing that FGF21 can protect chondrocytes from apoptosis, senescence and extracellular matrix catabolism by up-regulating autophagic flux, thereby alleviating the progression of osteoarthritis (180). This suggests that when exploring the impact of fasting on osteoarthritis, it is necessary to comprehensively consider its possible dual effects. Preclinical studies on the effect of IF in OA are presented in Table 1.

**TABLE 1:** Preclinical studies on the effect of IF in OA.

Model	Intervention	Duration	Subject	Mechanism	Results/conclusion	Reference
DMM OA	One, two, four, or six cycles of intermittent fasting (ADF for 1 week and then refeeding for another week)	12 weeks	C57BL/6 mice; Npy*^fl/fl^* mice	Osteocyte NPY is a key factor in the pathogenesis of osteoarthritis, and IF can reduce the expression level of NPY in osteocytes.	IF effectively mitigates aging-induced osteoarthritic phenotypes in DMM mice by enhancing physical function, reducing pain, stabilizing subchondral bone, preserving cartilage, suppressing osteoclastogenesis and inflammation, and inhibiting CGRP+ sensory nerve sprouting.	(162)
DMM OA	The mice were fasted for 24 h thrice a week (on days 2, 4, and 6), followed by *ad libitum* re-feeding of a standard solid diet for 1 week	2 months	C57BL/6 mice	Protect cartilage by activating OPTN-related mitophagy pathway.	IF reduces chondrocyte apoptosis and alleviates OA symptoms, including improvement in joint damage, increased pain threshold, and enhanced motor ability.	(23)
MIA OA	Rats had high-fat or high-protein for 3 h at the beginning of the dark cycle (19:00 to 22:00 h): 3-h feeding period	4 weeks	Rat	IF combined with high-protein diet can alleviate osteoarthritis symptoms by reducing hippocampal amyloid-β deposition and pro-inflammatory cytokine expression.	The combined intervention of IF and high-protein diet effectively alleviated the symptoms of AD and OA by increasing lean body mass, reducing inflammation, and improving metabolic status.	(174)
/	Alternating serum deprivation every hour (cyclical nutrient deprivation) in pyruvate and glucose-free medium	6 h	MC3T3 calvarial osteoblastic cell line	Cyclical nutrient deprivation can enhance mitochondrial function in osteogenic cells.	Simulated fasting can significantly improve mitochondrial function in osteoblasts, enhance the regenerative capacity of osteoprogenitors, and contribute to tissue repair.	(61)
/	/	/	Osteocytes isolated from sham, DMM, and DMM + IF mice	The culture medium of osteocytes from DMM+ *Ad libitum* mice had a much higher level of NPY compared with those from the sham-operated mice, whereas the peptide level of NPY from DMM + IF mice was profoundly decreased.	IF effectively inhibited these NPY-mediated pathological processes by reducing NPY expression levels in osteocytes.	(162)
/	Elevated FGF21 induced by food-restricted diet	/	Chondrocyte	Fasting induces an increase in the expression of FGF21 in the liver. FGF21 inhibits the proliferation and differentiation of chondrocytes by binding to FGFR1, FGFR3, and β-klotho, and reduces the binding of growth hormone to chondrocytes, thereby exerting an inhibitory effect on bone growth.	Fasting induces an increase in the expression of FGF21 in the liver, thereby exerting an inhibitory effect on bone growth.	(178, 179)

IF, intermittent fasting; OA, osteoarthritis; DMM, destabilization of the medial meniscus; MIA, monosodium iodoacetate; NPY, neuropeptide Y; OPTN, optineurin; AD, Alzheimer’s disease; FGF, fibroblast growth factor; FGFR, fibroblast growth factor receptor; CGRP, calcitonin gene related peptide.

## 6 Clinical evidence

### 6.1 Observational studies

Population cohort studies have reported subjective improvement in joint symptoms among individuals engaging in fasting practices driven by cultural or religious reasons, such as Ramadan fasting (62). However, these observational studies typically lacked rigorous control of confounding factors and objective evaluation of joint structural changes. Additionally, a multicenter cross-sectional study (181) indicated that fasting was relatively well-accepted by OA patients, particularly among those with hand OA, who reported overall improvement and pain relief following fasting practices. In a prospective exploratory observational study involving 125 OA patients (182), patients started fasting on the second day. It is usually scheduled for at least five consecutive days and a maximum of 12 days, with the length depending on the individual constitution of the patient and the regulation of the inpatient stay according to diagnosis and disease severity. During fasting, only natural juices, unsweetened herbal teas, and water are consumed. The daily caloric intake is between 200 and 300 calories. The study demonstrated that fasting significantly improved overall symptoms (mean WOMAC index reduction of 14.8 points), alleviated pain (mean NRS pain score reduction of 2.7 points), and reduced analgesic medication usage, with 36% of participants decreasing analgesic doses, discontinuing medication, or switching to herbal treatments. Furthermore, fasting enhanced patients’ quality of life (mean WHO-5 score increase of 4.5 points), reduced anxiety and depressive symptoms (mean reductions of 2.1 and 2.3 points in HADS-A and HADS-D, respectively), and facilitated weight loss and blood pressure reduction. Researchers believe that short-term fasting can be used as an adjunct to multimodal therapy to alleviate OA symptoms by rapidly reducing inflammation and weight. This prospective study provides direct human evidence for the use of IF in OA. However, it should be noted that this plan has a high intensity and should not be implemented frequently for a long time. Its long-term effectiveness and safety still need to be evaluated. Collectively, these findings indicate that extended fasting periods within multimodal therapeutic strategies may positively affect pain, functional status, and quality of life in knee and hip OA patients, although future randomized controlled trials are necessary to confirm these observations. Another outpatient fasting intervention study (183) lasting 2 weeks evaluated effects on pain, general health status, and joint function in OA patients. The study consisted of a 3-day preparatory period, an 8-day fasting period (approximately 300 kcal/day), and a 4-day recovery phase, with follow-ups at 4 and 12 weeks post-intervention. Results showed significant reductions in pain intensity during the fasting period and throughout the entire study duration, as well as improvements in joint function and overall health status. Analgesic medication use was also significantly reduced during fasting. These studies collectively provide preliminary evidence supporting the beneficial effects of fasting interventions for OA symptoms and suggest outpatient fasting under medical supervision may offer therapeutic potential for patients with OA.

### 6.2 Small-scale clinical trials and preliminary findings

Currently, direct clinical trials evaluating IF specifically for OA patients remain limited, though some related studies provide indirect evidence supporting its potential benefits. Extensive weight-loss clinical trials have clearly established the significance of weight reduction in improving OA symptoms (184, 185). While traditional weight-loss approaches rely heavily on continuous calorie restriction, IF serves as an alternative dietary strategy that demonstrates comparable weight-loss efficacy and adherence in obese populations (173). It is reasonable to hypothesize that IF-induced weight reduction would yield similar symptom relief in overweight OA patients (150). Meanwhile, studies have shown that IF can potentially treat different types of chronic pain through various mechanisms, providing a new non-invasive treatment approach for chronic pain management (18). A study proposed that decreased fasting insulin levels might play a crucial role in alleviating insulin resistance and inflammation in cartilage tissues (186). Concurrently, IF improved patients’ mood and sleep quality, potentially enhancing participation in rehabilitative exercises (187). Although this study was limited by its small sample size, these preliminary results highlight the need for larger, rigorous trials assessing the efficacy of IF in OA symptom management. A single-center, prospective, non-controlled observational study (188) involving 37 OA patients demonstrated that IF significantly improved joint pain, physical function, and quality of life. Notably, these clinical benefits were not only observed during the fasting period but also persisted during the post-fasting follow-up, suggesting that IF may confer sustained therapeutic effects in OA. Interestingly, the intervention had no significant impact on serum levels of N-ε-(carboxymethyl)-lysine (CML) or soluble receptor for advanced glycation end products (sRAGE), indicating that its benefits may not be mediated through modulation of these biochemical markers. The fasting protocol consisted of three distinct phases: a preparatory phase involving a low-calorie (∼ 800 kcal/day) lacto-vegetarian diet for 3 days; a fasting phase with severe caloric restriction (∼ 300 kcal/day) for 8 days; and a refeeding phase over 4 days with a stepwise increase in caloric intake (800, 1000, 1200, and 1600 kcal/day). During the fasting phase, intake of caffeine, alcohol, and nicotine was prohibited, and patients were encouraged to consume more than 2.5 liters of fluids daily. Researchers concluded that short-term fasting could serve as an adjunctive component within multimodal OA therapies, providing rapid reductions in inflammation and body weight to alleviate joint symptoms. However, the intensity of such fasting protocols may not be suitable for frequent or prolonged use, and long-term efficacy and safety warrant further investigation. A randomized controlled trial involving 32 patients with OA or chronic knee pain at risk of OA assessed the feasibility and acceptability of dietary interventions combined with relaxation and guided imagery therapy (189). Participants were allocated to three groups: IF, glucose-management, or normal-diet controls. Over a period of approximately 2 weeks, participants completed four experimental sessions. Those assigned to IF group were instructed to fast for 16 h prior to sessions 2–4. Specifically, they were asked to refrain from consuming any food or caloric beverages after 6:00 or 7:00 p.m. on the evening preceding each session, allowing only non-caloric drinks or black coffee during the fasting window. On non-session days, participants were advised to maintain their habitual dietary patterns. Individuals in the glucose administration group were instructed to fast for 2 h before sessions 2–4, while following their usual diet on all other days. Participants in the normal diet control group were asked to maintain their regular eating habits throughout the entire study duration. Compliance was high across all groups, with both IF and glucose-management groups achieving expected blood glucose ranges. Although no significant differences in experimental pain measures or mood and cognitive assessments were observed among the groups, the study importantly demonstrated that dietary interventions—IF and glucose management—are both feasible and acceptable adjuncts to chronic pain treatment when combined with relaxation and guided imagery. Moreover, several studies focusing on RA support potential fasting-related benefits for inflammatory joint disorders (190). For example, the “To eat or not to eat” trial compared a 7-day water-porridge fast followed by a plant-based diet against a purely anti-inflammatory diet in RA patients. Although there were no significant differences in disease activity scores between groups, fasting patients reported short-term improvement in joint pain and morning stiffness. Given the tolerability and symptom improvements observed in RA fasting studies, researchers suggest exploring similar approaches in OA populations.

A crossover study involving 14 patients with hand, hip, or knee OA reported that fasting combined with caloric intake devoid of protein may impair cartilage sulfation, potentially influencing OA pathogenesis (191). In this study, participants refrained from food, medications, vitamins, and smoking from 10:00 p.m. the night prior. On one test day, they continued fasting for an additional 3 h in the morning. On a separate day, following the same overnight fast, they ingested 75 grams of glucose at midnight and again remained fasted for the subsequent 3 h. Serum sulfate levels were measured across both conditions. Results showed a mean 9.3% reduction in serum sulfate levels after fasting alone, which was further exacerbated to an average 18.9% decrease following glucose ingestion, indicating that both fasting and postprandial glycemic shifts may suppress systemic sulfate availability. As sulfate is essential for proteoglycan sulfation in cartilage, these findings suggest that prolonged fasting and insufficient protein intake may negatively affect cartilage integrity. The study underscores the importance of adequate protein consumption and cautions against extended fasting regimens in individuals with OA.

Overall, large-scale randomized controlled trials specifically evaluating IF for OA are currently lacking. However, combined evidence from weight-loss studies, observational fasting trials, and investigations in related inflammatory joint diseases collectively support IF’s potential to relieve joint symptoms and reduce inflammation markers (18, 48, 192). Clinical studies on the effect of IF in OA are presented in Table 2.

**TABLE 2:** Clinical studies on the effect of IF in OA.

Study design	Intervention	Sample size	Duration	Research subject	Detection method	Finding	Conclusion	Reference
A multicentre cross-sectional study	A self-administered questionnaire explored dietary practices and patients’ perceived effects of diet, foods and beverages on symptoms.	Total 392 RA: 123 (female = 104, male = 19) axSpA: 161 (female = 67, male = 94) Hand OA: 108 (female = 91, male = 17)	Data collection period: January 2019 to May 2020	Patients with RA, axSpA or hand OA	The primary outcome was the proportion of patients who were following or had followed one or more diets of whatever duration over their lifetime in the total sample and for each RMD. Secondary outcomes were: (1) the type of diet followed for each RMD; (2) patients’ opinions about the positive or negative effect of dieting on pain, fatigue or general condition as well as the effect of particular foods and/or beverages on pain; (3) factors associated with dieting; and (4) factors associated with patients’ views on the effect of food or drinks on pain.	The detox/fasting (low glycemic and energy intake, interspersed with periods of normal eating) diet (26.2%) were the most popular among hand OA patients. The median duration of diets was 12 months (IQR: 4.0–30.0). The detox/fasting diet did not modify pain for 60.9% of patients but improved the general condition for 73.3%.	Fasting has a certain level of acceptance among patients with hand OA, and they believe that fasting has improved their overall condition and alleviated their pain to some extent.	(181)
A prospective exploratory observational study	Fasting was the only common intervention for all patients, being performed as part of a multimodal integrative treatment program, with a daily caloric intake of <600 kcal.	125 (female = 107, male = 18)	It is usually scheduled for at least 5 consecutive days and a maximum of 12 days (7.7 ± 1.7 days)	All patients with OA as their main diagnosis	Questionnaire assessments (WOMAC Index, HADS scale, MAAS scale, WHO-5 questionnaire, NRS pain score); measurement of physiological parameters; and recording of medication use	An amelioration of overall symptomatology (WOMAC Index core: −14.8 ± 13.31; *p* < 0.001; *d* = 0.78) and pain alleviation (NRS pain: −2.7 ± 1.98, *p* < 0.001, *d* = 1.48). Pain medication was reduced, stopped, or replaced by herbal remedies in 36% of patients. Increased quality of life (WHO-5: +4.5 ± 4.94, *p* < 0.001, *d* = 0.94), reduced anxiety (HADS-A: −2.1 ± 2.91, *p* < 0001, *d* = 0.55) and depression (HADS-D: −2.3 ± 3.01, *p* < 0.001, *d* = 0.65), and decreases in body weight (−3.6 kg ± 1.65, *p* < 0.001, *d* = 0.21) and blood pressure (systolic: −6.2 ± 15.93, *p* < 0.001, *d* = 0.43; diastolic: −3.7 ± 10.55, *p* < 0.001, *d* = 0.43)	Prolonged fasting, as part of a multimodal integrative treatment approach, has a positive impact on improving the quality of life, pain, and disease-specific functional parameters in patients with OA of the of the lower extremities.	(182)
Uncontrolled clinical study	Underwent ambulant fasting therapy for 2 weeks with 3 pre-fast days, 8 fast days (300 kcal) and 4 re-feed days as well as follow-up 4 and 12 weeks afterward.	30 (female = 22, male = 8)	2 weeks	OA (Kellgren stages I–III) of the hand (*N* = 10), hip (*N* = 8) and knee (*N* = 12)	VAS pain score: joint pain with activity, with start of walking, at rest; pressure pain threshold; articular function; health-related quality of life (SF-36); WOMAC Index; pain DETECT© questionnaire (Pfizer); analgesics; weight; BMI; waist circumference; blood pressure; pulse and a variety of serological parameters.	Pain, state of health, and articular function improved significantly; significant reduction in weight, BMI, and waist circumference during fasting and over the complete course of the study; analgesics could be reduced. No abnormalities in autonomous, metabolic, or blood parameters were observed.	Medically supervised fasting can have a positive impact on the symptoms of patients with moderate OA.	(183)
A single-center, prospective, non-controlled observational study	Therapeutic fasting was performed in an outpatient setup and comprised preparation, fasting, and refeeding. Three preparatory lacto-vegetarian diet days (∼ 800 kcal/day) were followed by 8 fasting days (∼ 300 kcal/day) and 4 re-feeding days with gradually increasing calorie intake (800, 1,000, 1,200, and 1,600 kcal/day). Caffeine, alcohol, and nicotine were not allowed during the intervention period. Patients were encouraged to drink > 2.5 L of liquids daily.	37 (female = 34, male = 3)	8 days	Patients with unilateral or bilateral OA diagnosed by EULAR criteria	The serum levels of CML and sRAGE, as well as the sRAGE/CML ratio. Clinical parameters such as VAS, WOMAC, and SF-36.	The CML levels did not significantly change from baseline to the end of intervention (Δ = −25.6 ± 92.2 ng/ml; *p* = 0.10). In contrast, the sRAGE levels (Δ = −182.7 ± 171.4 ng/ml; *p* < 0.0001) and the sRAGE/CML ratio (Δ = −0.4 ± 0.6; *p* < 0.001) significantly decreased, but they returned to baseline levels 4 weeks after the end of fasting. The BMI [baseline: 28.7 (6.8), end of fasting: 27.2 (6.5), follow-up: 27.2 (6.5)], waist circumference [Baseline: 97.0 (15.5), end of fasting: 93.4 (15.6), follow-up: 92.3 (15.4)], systolic blood pressure [baseline: 129.3 (16.8), end of fasting: 122.8 (16.0), follow-up: 121.8 (14.1)] and diastolic blood pressure [baseline: 83.6 (11.0), end of fasting: 80.8 (9.2), follow-up: 78.2 (11.7)] significantly decreased at the end of fasting and at follow-up (4 weeks after the end of fasting) in comparison to baseline. VAS improved from baseline to the end of fasting (−2.7 ± 1.9) and at follow-up (−2.2 ± 2.0). The same improvement was observed for start-up pain (−1.5 ± 1.7 at the end of fasting; −1.5 ± 2.0 at follow-up), movement-induced pain (−1.7 ± 1.9 at the end of fasting; −1.9 ± 2.3 at follow-up), and for pain at rest (−0.8 ± 1.9 at the end of fasting; −1.0 ± 1.8 at follow-up). SF-36 improved for the PCS (3.0 ± 4.4 at the end of fasting; 5.4 ± 5.6 at follow-up) and for the MCS (1.8 ± 5.2 at follow-up). WOMAC improved in all three categories: pain (−17.6 ± 13.6 at the end of fasting; −14.0 ± 13.6 at follow-up), stiffness (−19.8 ± 19.7 at the end of fasting; −14.0 ± 20.7 at follow-up), and function (−14.0 ± 13.1 at the end of fasting; −11.8 ± 13.7 at follow-up).	Fasting resulted in a significant but non-sustained reduction of sRAGE levels and the sRAGE/CML ratio in OA, while the CML levels did not change. Improvement in clinical endpoints of OA does not correlate with changes in CML or sRAGE.	(188)
A pilot randomized controlled trial	The IF group (*n* = 11, fast for 16 h prior to sessions 2–4). The glucose administration group (*n* = 11, fast for 2 h prior to sessions 2–4. At the beginning of sessions 2–4, blood glucose level was assessed, then participants consumed 30 g of a pharmaceutical grade liquid glucose). The normal-diet group (*n* = 10).	32 (female = 14, male = 18)	14 days	Adults with chronic knee pain due to or at risk for OA, with a duration of at least 3 months.	Measures described are limited to those addressing feasibility and acceptability.	Participants completed all assessment procedures. No adverse events were reported. Blood glucose levels in both the IF group and the glucose administration group after the intervention were within the expected ranges, although no significant differences were observed between the groups in experimental pain testing as well as affective and cognitive assessments.	This study demonstrates the feasibility and acceptability of combining IF and glucose administration with relaxation and guided imagery therapy in patients with chronic knee pain.	(189)
A cross-over study	Patients fasted from 22:00 h the preceding night, without drugs, vitamins or smoking. At time 0 in the morning they continued their fast for 3 h, and after an overnight fast on another day took 75 g of glucose at time 0 with no additional ingestion for 3 h.	14 (female = 11, male = 3)	3 h	Adults with hand, hip, or knee OA	Measure serum levels of sulfate during 3 h of fasting or glucose ingestion after overnight fasts	A 3-h continuation of fasting caused a marked reduction in serum sulfate levels, whereas ingestion of 75 g of glucose in the absence of protein resulted in doubling the reduction.	Fasting and the intake of protein-free calories may lead to a period of chondroitin under sulfation that can affect OA, thereby exacerbating its progression.	(191)

IF, intermittent fasting; OA, osteoarthritis; RA, rheumatoid arthritis; axSpA, axial spondyloarthritis; RMDs, rheumatic and musculoskeletal diseases; HADS, hospital anxiety and depression scale; MAAS, mindful attention awareness scale; WHO-5, the World Health Organization-Five Well-Being Index; NRS, numerical rating scale; BMI, body mass index; CML, N-ε-(carboxymethyl)-lysine; sRAGE, soluble receptor for advanced glycation end products; VAS, visual analog scale; SF-36, 36-Item Short-Form Health Survey; WOMAC, Western Ontario and McMaster Universities Arthritis Index; PCS, physical component scale; MCS, mental component scale.

Despite recent progress, research on the IF-OA relationship still faces limitations. The number of randomized controlled trials involving IF interventions in OA patients remains limited, often characterized by small sample sizes, short durations, and absence of long-term follow-up. Future large-scale, multicenter randomized controlled trials are required to validate IF’s clinical effectiveness on OA symptoms, radiographic outcomes, and cartilage biomarkers (e.g., type II collagen C-terminal peptide, cartilage oligomeric matrix protein). Additionally, further investigation should address the safety and compliance of IF regimens in older adults or those with comorbidities such as diabetes or cardiovascular disease.

## 7 Practical considerations and future directions

### 7.1 Adherence and safety

Research indicates that IF rarely produces adverse gastrointestinal, neurological, hormonal, or metabolic outcomes (39, 193, 194). For OA patients, the selection of an appropriate IF regimen should be based on individual lifestyle patterns and compliance capabilities. Patients with regular daily routines may find TRF-such as skipping breakfast and limiting eating to between noon and 8:00 PM-relatively easy to incorporate, as it aligns closely with circadian rhythms and reduces perceived hunger. For younger and middle-aged patients needing to accommodate family meals or social interactions, the 5:2 diet, which allows the consumption of approximately 500–600 kcal of simple foods on two fasting days per week, offers greater flexibility while maintaining normal eating on non-fasting days. Conversely, alternate-day fasting, despite significant clinical benefits demonstrated in research, is associated with fluctuating energy states that may disrupt work and daily routines, potentially limiting its suitability for general OA populations. Moreover, patients requiring medication at specific times may face difficulties adhering to certain time-restricted eating schedules. Thus, these individuals should collaborate with their clinicians to develop a fasting regimen that integrates effectively with their medication protocols (39). Patient adherence is critical for achieving therapeutic outcomes with IF. Evidence suggests that patients who consistently adhere to IF experience significantly better improvements in pain relief and functional outcomes after 3 months compared to those who discontinue early (18). Therefore, healthcare providers should offer comprehensive health education and ongoing support to patients by clearly explaining the scientific basis and potential benefits of IF, assisting with detailed meal planning, providing practical strategies to manage potential discomfort such as hunger and dizziness, and scheduling regular follow-ups to encourage continued adherence. For patients with cognitive impairment or poor self-control, IF implementation is not advisable, as it may elevate the risk of nutritional deficiencies or other health complications.

### 7.2 Monitoring potential risks

Although most studies have concluded that IF is safe for metabolically healthy adults (18), caution remains necessary for OA patients, who are often older adults with comorbidities. Several potential risks warrant consideration. First, hypoglycemia and hypotension: fasting may lead to reduced blood glucose, particularly in patients receiving diabetes medications; orthostatic hypotension can also occur due to prolonged periods without food intake. Patients should be instructed on blood glucose monitoring and, when necessary, consume small amounts of sugary beverages to correct hypoglycemia during fasting, as well as advised on gradual posture changes to prevent dizziness. Second, gout: fasting elevates ketone levels and may transiently increase serum uric acid; therefore, patients with gout should adopt IF carefully under medical supervision (158). Third, gallstones: rapid weight loss and extended fasting periods may increase the risk of bile stasis, and previous studies have documented higher gallstone incidences following significant rapid weight reductions (195). OA patients with a history of gallbladder disease require regular monitoring. Fourth, IF is contraindicated in children under 12 years, pregnant or breastfeeding women, as safety studies are currently lacking in these groups. Fifth, dietary composition warrants critical attention. Protein malnutrition and inadequate vitamin intake represent key nutritional risks during IF regimens (196). Protein is essential for preserving muscle mass and joint integrity, while vitamins support immune competence, cellular repair, and metabolic homeostasis. In addition, during IF, patients also need to maintain the health of their gut microbiota through fiber rich foods to indirectly support joint function, while ensuring calcium intake to protect bone health (39). It is worth noting that insufficient caloric intake, dehydration, and elevated stress hormone levels—commonly associated with restrictive diets—can lead to transient symptoms such as headaches, fatigue, dizziness, and nausea. However, these effects are typically alleviated upon refeeding (56). Overall, IF should be practiced under medical supervision, including clear emergency guidelines, such as immediate cessation of fasting and seeking medical attention if severe adverse events occur. With proper regimen selection and clinical guidance, most patients can successfully navigate the adaptation phase and safely benefit from IF.

### 7.3 Patient selection and individualized protocols

Based on existing evidence, overweight or obese patients with KOA may represent the primary candidate population for IF interventions. Weight reduction in these individuals clearly improves OA outcomes, and IF offers an alternative weight-loss approach that many patients find more sustainable than daily calorie restriction (173). For OA patients who have a normal body weight but exhibit metabolic disturbances, such as prediabetes or hyperinsulinemia, IF might also confer benefits by improving metabolic profiles; however, careful nutritional assessment is necessary to avoid excessive weight loss. Individualization of IF protocols is especially important in older adults (≥65 years) with OA, given the higher risk of sarcopenia. These patients should ensure adequate daily protein intake, preferably consuming high biological-value proteins within their eating window to preserve muscle mass (197). Furthermore, patients with concurrent diabetes and those receiving hypoglycemic medications should consult their physicians before initiating IF to adjust medication dosages and prevent hypoglycemic episodes. In clinical practice, the implementation of IF should adhere to a principle of “start low, monitor closely, and adjust gradually.” For example, initiating a 12-h daily fast (from 8 PM to 8 AM) and progressively extending fasting duration or transitioning to a 5:2 regimen should be based on patient tolerance and observed efficacy.

### 7.4 Combining IF with other interventions

The synergistic potential of combining IF with exercise therapy, pharmacological treatments (such as non-steroidal anti-inflammatory drugs [NSAIDs] or slow-acting osteoarthritis agents), and joint-protective nutrients (e.g., omega-3 fatty acids, vitamin D) is an emerging area of interest (10). A multimodal therapeutic approach is recommended, as IF does not conflict with existing treatments but rather complements exercise and nutritional supplementation strategies. First, IF aligns well with anti-inflammatory dietary concepts (18); thus, dietary patterns enriched in omega-3 fatty acids, dietary fiber, and antioxidants can be integrated alongside IF to enhance anti-inflammatory benefits. Second, moderate strength training and aerobic exercise during IF periods may further promote fat utilization and muscle protein synthesis, significantly improving joint function. A community-based study demonstrated that combining dietary restriction with exercise resulted in a 50% greater reduction in knee pain compared to dietary restriction alone, suggesting additive benefits for mobility improvement (184). Therefore, IF should be promoted as part of a comprehensive lifestyle intervention plan rather than as an isolated or replacement therapy for OA patients. Additionally, IF may beneficially interact with supplements such as probiotics or prebiotics, which could further optimize gut microbiota composition, or protein hydrolysis enzyme inhibitors (e.g., quercetin) that potentially amplify fasting-induced metabolic effects. These interactions warrant investigation in future studies. It is important to emphasize that IF cannot replace surgical interventions such as joint arthroplasty in advanced OA cases; however, it could play an essential role in perioperative weight management and metabolic optimization, improving surgical tolerance and postoperative recovery outcomes. Caution is warranted when combining IF with exercise interventions. The fasting window may predispose individuals to hypoglycemia and insufficient energy availability, potentially impairing physical performance and post-exercise recovery (198). This concern is particularly relevant for individuals with irregular daily routines or those requiring frequent caloric intake to sustain metabolic demands. Adhering to fixed schedules for both feeding and training may thus pose practical challenges. To mitigate these risks, it is advisable to adapt exercise timing in alignment with the fasting cycle, avoiding high-intensity workouts during periods of low blood glucose to optimize both safety and efficacy.

### 7.5 Limitations and future research priorities

Limitations: Significant physiological, metabolic, and lifespan differences between animal models (such as mice and rats) and humans must be acknowledged (56). For example, mice exhibit markedly higher metabolic rates than humans and may respond differently to nutritional changes. Furthermore, disease models in animals may not fully replicate the complexity of human conditions. In human studies, adherence to IF protocols may influence outcomes, and individual variability in response to IF is common (189). Some studies also exhibit methodological limitations (182), such as the absence of appropriate control groups, lack of detailed data on participants’ overall dietary behaviors, and insufficient tracking of dietary changes during follow-up. These factors hinder accurate attribution of observed effects to IF alone, as changes in post-discharge diet may also play a role. Moreover, long-term therapeutic fasting is often combined with exercise or mind–body interventions to alleviate hunger or adverse symptoms, potentially introducing confounding variables and masking possible side effects of IF (199). In addition, most human trials to date involve relatively short intervention periods, providing limited evidence regarding the long-term health effects of IF.

Despite preliminary evidence supporting the potential of IF for OA management, several important scientific questions remain to be addressed:

(1) Clinical efficacy evidence: There is an urgent need for large-scale randomized controlled trials (RCTs) to rigorously evaluate the effects of IF on OA-related pain, functional outcomes, and radiographic progression, particularly in comparison with traditional continuous calorie restriction diets. Such trials should include distinct patient subgroups (e.g., obese vs. non-obese OA patients) to identify populations that could derive the greatest benefit from IF.
(2) Mechanistic biomarkers: Future investigations should delve deeper into the molecular mechanisms by which IF influences OA. Advanced technologies such as single-cell sequencing and tissue organoids can be employed to elucidate multidimensional effects of IF on chondrocytes, osteocytes, and synovial cells, with special emphasis on novel neuroendocrine pathways like NPY. Imaging techniques, such as MRI T2 mapping, should be utilized to quantitatively assess cartilage quality and structural changes, while biochemical markers in serum and synovial fluid (e.g., type II collagen C-terminal peptide, cartilage oligomeric matrix protein) could serve as sensitive indicators of IF intervention efficacy (182).
(3) Optimization of IF protocols: Comparative analyses of different IF regimens (e.g., alternate-day fasting vs. 16:8 fasting), varying fasting durations (such as 14 vs. 18 h daily), and their respective impacts on OA outcomes are necessary to define the optimal fasting strategy. Additionally, the relationship between intervention duration and effectiveness should be explored to ascertain whether sustained adherence yields cumulative benefits.
(4) Combined interventions and functional recovery: Evaluating the potential synergy of IF with exercise rehabilitation programs and pharmacological therapies (e.g., analgesics, disease-modifying osteoarthritis drugs) could reveal important interactions, such as reducing reliance on NSAIDs or enhancing the efficacy of joint-protective supplements (e.g., glucosamine).
(5) Special populations and risk management: Special attention is needed regarding safety, efficacy, and nutritional status monitoring of IF interventions among older OA patients and those with comorbid conditions, including metabolic syndrome, diabetes, and osteoporosis risk, to minimize adverse outcomes.
(6) Alternative strategies: For patients unable to adhere to dietary changes, pharmaceutical mimetics of IF, such as low-dose mTOR inhibitors or AMPK activators, could be explored to stimulate similar beneficial metabolic pathways without the necessity of fasting (23).

## 8 Conclusion

The complex pathogenesis of OA limits the effectiveness of conventional therapies targeting single mechanisms, highlighting the need for systemic therapeutic strategies. IF, as a systemic dietary intervention, offers novel therapeutic potential for OA prevention and management. This review summarizes recent mechanistic and preliminary clinical evidence demonstrating that IF exerts multifaceted effects addressing critical pathological aspects of OA. Animal studies have shown that IF alleviates cartilage degradation, inhibits synovial inflammation, and corrects abnormal bone remodeling processes, highlighting emerging molecular mechanisms such as NPY signaling (162). Preliminary clinical observations further suggest that IF can feasibly improve pain and functional outcomes in OA patients (182). Nonetheless, current evidence primarily derives from preclinical and small-scale clinical studies; therefore, robust, long-term clinical trials are essential to definitively establish the efficacy and safety of IF for OA patients. Practically, IF protocols must be individually tailored according to patient characteristics and integrated into comprehensive intervention programs alongside weight management, exercise, and other lifestyle modifications. With increasing mechanistic insights and clinical data accumulation, IF is poised to evolve from a complementary lifestyle modification to an integral component of holistic OA management, representing a promising non-pharmacological and non-surgical therapeutic option. In conclusion, IF demonstrates considerable potential in OA prevention and treatment, offering new hope in addressing the growing global burden of osteoarthritis.
